# Change in global transmission rates of COVID-19 through May 6 2020

**DOI:** 10.1371/journal.pone.0236776

**Published:** 2020-08-06

**Authors:** Ethan Obie Romero-Severson, Nick Hengartner, Grant Meadors, Ruian Ke

**Affiliations:** 1 Theoretical Biology and Biophysics Group, Los Alamos National Laboratory, Los Alamos, NM, United States of America; 2 Computational Physics Group, Los Alamos National Laboratory, Los Alamos, NM, United States of America; University of Leeds, UNITED KINGDOM

## Abstract

We analyzed COVID-19 data through May 6th, 2020 using a partially observed Markov process. Our method uses a hybrid deterministic and stochastic formalism that allows for time variable transmission rates and detection probabilities. The model was fit using iterated particle filtering to case count and death count time series from 55 countries. We found evidence for a shrinking epidemic in 30 of the 55 examined countries. Of those 30 countries, 27 have significant evidence for subcritical transmission rates, although the decline in new cases is relatively slow compared to the initial growth rates. Generally, the transmission rates in Europe were lower than in the Americas and Asia. This suggests that global scale social distancing efforts to slow the spread of COVID-19 are effective although they need to be strengthened in many regions and maintained in others to avoid further resurgence of COVID-19. The slow decline also suggests alternative strategies to control the virus are needed before social distancing efforts are partially relaxed.

## Introduction

Since its initial outbreak in Wuhan, China in late 2019 and early 2020 [1], COVID-19 caused repeated rapid outbreaks across the global from February through April 2020. The extremely rapid spread of COVID-19 in China [2] does not appear to be an anomaly: the disease has shown a short doubling time (2.4-3.6 days) outside of China as well [3]. As of May 21, 2020, the virus caused 5,034,458 reported infections, and 328,730 deaths globally [4]. In response, most affected countries/regions have implemented strong social distancing efforts, such as school closures, working-from-home, shelter-in-place orders. As a result, the spread of COVID-19 slowed down substantially in some countries [5], leading to a flattening of the epidemic curve. As social distancing induces high costs to both society and individuals, plans to relax social distancing are discussed. However, changes in both the transmission rates and detection probabilities over time coupled with stochasticity due to reporting delays makes differentiating between truly subcritical dynamics and simply reduced transmission difficult.

In this report, we developed a deterministic-stochastic hybrid model and fitted the model to case incidence and death incidence time series data from 55 countries. Following the approach suggested by King et al. [6], we use a (partially) stochastic model and base our estimates on incidence rather than cumulative incidence data. Using both case count and death count of data allowed us to disentangle changes in surveillance intensity from changes in transmission [3]. We found evidence for large decreases in the country-level transmission rates, in several of the worst-affected countries. Importantly, using data up to May 6, 2020, we computed 99% confidence intervals to test whether or not the data were consistent with subcritical dynamics (i.e. the reproductive number *R* was below 1 on May 6, 2020). Most countries showed large decreases in transmission rates over time, and more than half of studied countries have transmission rates below the epidemic threshold. On the other hand, many countries still appear to be showing rapid exponential growth. Given its highly contagious nature, COVID-19 can spread rapidly when strong social distancing measures are lifted, even partially [3]. Alternative strategies that can effective control the virus are needed when social distancing measures are relaxed.

## Materials and methods

### Data

Case count and death data were downloaded from the Johns Hopkins GitHub repository (https://github.com/CSSEGISandData/COVID-19) through May 6, 2020. Data included aggregate counts of reported cases and deaths at the country level and contained no identifying information. Any country in the data that had more than 2000 cumulative cases and 100 deaths by May 6, 2020 was included. To minimize the effect of repatriated cases we started each time series on the first day when the cumulative number of cases exceeded 100. All data processing and model fitting were otherwise done on the incidence scale. To address obvious bulk-reporting issues in the data (e.g. sudden zeros in the data followed by very large numbers), we smoothed the data using Tukey’s 3-median method. Because several countries had days with a single death surrounded by days with no deaths, which the smoothing method would set to zero, days with a single death were not replaced with smoothed values. The original data and the smoothed data used for estimation are shown in S1 Fig in S1 File.

### Model

We model the spread of COVID-19 as a partially observed Markov process with real-valued states *S* (susceptible), *E* (exposed), *I* (infected), and *R* (removed) to describe the latent population dynamics, and integer-valued states *C*_0_ (to be counted), *Y*_1_ (counted cases), *D*_0:3_ (dying), and *Y*_2_ (counted deaths) to model sampling into the data. We use multiple states to model the counted deaths to produce an Erlang distribution with mean 21 days and standard deviation 11 days in the time to death based on previous estimates of the time to death [7]. The model and all of its parameters (Table 1) have time units of days. The latent population model is governed by the following ordinary differential equations,

$$\begin{array}{cc} \frac{dS}{dt} & =-\chi \left(t\right)I\frac{S}{N},\\ \frac{dE}{dt} & =\chi \left(t\right)I\frac{S}{N}-\lambda_{EI}E,\\ \frac{dI}{dt} & =\lambda_{EI}E-\lambda_{IR}I,\\ \frac{dR}{dt} & =\lambda_{IR}I, \end{array}$$

where *χ*(*t*) is the time-variable transmission rate and *N* = *S* + *E* + *I* + *R* is assumed to be fixed over the run of the model. λ_*EI*_ is the rate at which exposed persons become infectious and λ_*IR*_ is the rate at which cases recover (i.e. are either no longer infectious or die). At every time interval, we sample persons moving from the *E* to *I* states into a stochastic arm of the model that is used to calculate the likelihood of the data.

**Table 1:** Table of key parameter values. The ‘Model’ column referrers to the best model defined in the model selection procedure. The values *r*_0_ and *r*_*f*_ are the initial and final exponential growth rates implied by the other model parameters. Note that values of *r* assume a fully susceptible population to ensure that the change in growth rates are comparable between countries. The term *I*(−21) indicates the number of infected persons 21 days prior to the first included observation. *χ*_0_, *χ*_*f*_, *r*_0_, *r*_*f*_, have day^−1^ units; *ρ*_0_ and *ρ*_*f*_ are probabilities. The error terms *ϵ*_1_ and *ϵ*_2_ are defined according to the definition of the overdispersion term used in the Negative Binomial distribution as implemented in R (i.e. in the limit as *ϵ* becomes large, the error becomes Poisson).

Country	Model	*I*(−21)	*r*_0_	*r*_*f*_	CI *r*_*f*_	*χ*_*f*_	CI *χ*_*f*_	*ρ*_0_	*ρ*_*f*_	*ϵ*_1_	*ϵ*_2_
Algeria	2	869	0.12	0.008	0.007, 0.013	0.111	0.109, 0.117	0.08	0.08	26.8	0.4
Argentina	1	34	0.25	0.008	0.008, 0.017	0.111	0.11, 0.123	0.16	0.16	18.6	131.8
Austria	2	23	0.22	-0.07	-0.072, -0.069	0.024	0.022, 0.025	0.25	0.25	45	882.2
Bangladesh	3	997	0.11	0.065	0.064, 0.066	0.197	0.195, 0.199	0.1	0.1	32.9	0.3
Belarus	1	992	0.05	0.044	0.014, 0.08	0.163	0.119, 0.223	0.79	0.79	0.2	1096.5
Belgium	3	101	0.2	-0.006	-0.009, -0.004	0.092	0.088, 0.095	0.05	0	23.4	5.4
Brazil	2	989	0.13	0.058	0.057, 0.059	0.185	0.183, 0.186	0.05	0.05	6.3	6
Canada	3	9	0.27	0.039	0.038, 0.04	0.156	0.154, 0.157	0.11	0	45.8	24.4
Chile	1	2	0.35	0.038	0.037, 0.039	0.154	0.153, 0.156	0.55	0.55	8	489.7
China	1	919	0.25	-0.069	-0.071, -0.067	0.025	0.023, 0.027	0.24	0.24	0.4	5.5
Colombia	1	6	0.32	0.035	0.027, 0.038	0.15	0.137, 0.154	0.18	0.18	14.1	314.3
Czechia	2	25	0.22	-0.066	-0.088, -0.054	0.027	0.009, 0.039	0.28	0.28	16.4	440.3
Denmark	2	750	0.1	-0.042	-0.048, -0.032	0.05	0.044, 0.061	0.19	0.19	7.4	56
Dominican Republic	1	524	0.13	0.016	0.015, 0.026	0.122	0.12, 0.136	0.24	0.24	16.5	41.9
Ecuador	1	724	0.15	0.044	0.025, 0.063	0.163	0.134, 0.194	0.13	0.13	0.3	0.2
Egypt	2	982	0.08	0.045	0.044, 0.046	0.165	0.163, 0.165	0.12	0.12	563.7	102
Finland	3	403	0.07	0.023	0.021, 0.024	0.131	0.128, 0.133	0.23	0.03	23.5	33.4
France	2	26	0.29	-0.097	-0.1, -0.095	0.002	<0.001, 0.004	0.07	0.07	3.6	2.7
Germany	3	2	0.33	-0.071	-0.076, -0.036	0.023	0.019, 0.057	0.38	0.02	22	11.2
Greece	2	942	0.07	-0.07	-0.1, -0.044	0.024	<0.001, 0.048	0.18	0.18	12.5	35.3
Hungary	2	930	0.08	-0.011	-0.019, -0.005	0.087	0.077, 0.093	0.08	0.08	22.3	568.4
India	3	18	0.23	0.041	0.04, 0.042	0.158	0.156, 0.16	0.19	0.05	31.8	5.9
Indonesia	2	983	0.09	0.017	0.012, 0.017	0.122	0.116, 0.123	0.07	0.07	51.4	2.4
Iran	1	2	0.52	-0.007	-0.008, -0.005	0.092	0.09, 0.093	0.15	0.15	2.9	8.7
Iraq	1	865	0.05	0.009	0.004, 0.014	0.112	0.105, 0.118	0.21	0.21	4.6	2.2
Ireland	2	131	0.17	-0.025	-0.03, -0.015	0.069	0.063, 0.081	0.12	0.12	16.6	8
Israel	2	1	0.28	-0.085	-0.097, -0.067	0.012	0.002, 0.026	0.66	0.66	18.2	102.8
Italy	2	990	0.18	-0.042	-0.043, -0.039	0.051	0.049, 0.054	0.06	0.06	17.7	1.2
Japan	1	877	0.02	0.054	0.053, 0.056	0.18	0.177, 0.183	0.18	0.18	2.4	5.1
Malaysia	2	1	0.35	-0.049	-0.056, -0.047	0.043	0.037, 0.045	0.54	0.54	14.3	5.2
Mexico	2	485	0.14	0.052	0.051, 0.062	0.175	0.174, 0.192	0.05	0.05	49.1	17.4
Moldova	3	29	0.17	-0.012	-0.017, 0.003	0.085	0.079, 0.103	0.3	0	35.4	173.5
Morocco	1	240	0.15	0.016	0.015, 0.019	0.121	0.12, 0.125	0.23	0.23	10.8	1.1
Netherlands	3	69	0.25	-0.046	-0.049, -0.045	0.046	0.043, 0.048	0.08	0.01	30.2	18.2
Norway	2	1	0.46	-0.03	-0.036, -0.024	0.063	0.058, 0.07	0.35	0.35	4	62.2
Pakistan	1	983	0.07	0.056	0.052, 0.063	0.183	0.176, 0.194	0.34	0.34	15.1	128.2
Panama	2	422	0.09	-0.032	-0.034, -0.012	0.061	0.059, 0.084	0.34	0.34	17.6	34177.8
Peru	1	326	0.13	0.097	0.097, 0.106	0.255	0.255, 0.271	0.1	0.1	5.6	13.3
Philippines	2	351	0.13	-0.015	-0.015, -0.013	0.082	0.081, 0.084	0.16	0.16	5.9	29.6
Poland	2	107	0.18	-0.024	-0.027, -0.023	0.071	0.068, 0.072	0.19	0.19	76.1	133.4
Portugal	2	111	0.19	-0.046	-0.05, -0.044	0.046	0.043, 0.049	0.22	0.22	12.6	262.6
Romania	2	67	0.19	-0.013	-0.019, -0.012	0.084	0.076, 0.085	0.15	0.15	66.2	70.8
Russia	2	90	0.15	0.034	0.033, 0.036	0.148	0.146, 0.15	0.43	0.43	65.4	2
Saudi Arabia	1	923	0	0.079	0.077, 0.082	0.222	0.218, 0.227	0.96	0.96	11.1	3.3
Serbia	2	268	0.09	-0.099	-0.1, -0.094	0.001	<0.001, 0.005	0.41	0.41	22.4	337
South Africa	1	5	0.28	0.034	0.024, 0.036	0.148	0.133, 0.151	0.5	0.5	3.2	39.6
South Korea	3	988	0.08	-0.034	-0.035, -0.033	0.059	0.059, 0.061	0.45	0.01	6	47.7
Spain	2	17	0.34	-0.082	-0.093, -0.067	0.014	0.005, 0.027	0.08	0.08	6.1	6.8
Sweden	3	446	0.13	0.048	-0.006, 0.056	0.169	0.093, 0.182	0.08	0.07	17.2	1
Switzerland	2	41	0.22	-0.069	-0.071, -0.067	0.024	0.023, 0.026	0.17	0.17	21.7	153.4
Turkey	2	492	0.18	-0.034	-0.035, -0.032	0.059	0.058, 0.061	0.22	0.22	17.2	2.4
Ukraine	2	716	0.09	-0.012	-0.014, 0.001	0.085	0.082, 0.101	0.33	0.33	50.3	83.9
United Arab Emirates	1	5	0.26	0.063	0.061, 0.084	0.193	0.19, 0.23	0.99	0.99	1.9	21.5
United Kingdom	3	23	0.3	-0.004	-0.004, -0.002	0.095	0.094, 0.097	0.05	0	31.3	5
US	3	22	0.29	0	-0.001, 0.001	0.1	0.098, 0.101	0.09	0.07	44.5	3.2

To relate the latent population model to data, we randomly sample individuals from the unobserved population into a stochastic process that models the random movement from infection to being either counted as an observed case or counted as an observed death. The number of persons sampled into the observation arm of the model over a time interval *dt* is given by *X* = (*X*_1_, *X*_2_, *X*_3_, *X*_4_) ∼ Multinomial(*g*(*E*λ_*EI*_ *dt*), {(*ρ*^*c*^(*t*)*ω*^*c*^, *ρ*^*c*^(*t*)*ω*, *ρ*(*t*)*ω*^*c*^, *ρ*(*t*)*ω*}) where *ρ*(*t*) and *ω* are the probabilities that an infected person will be counted or die respectively, *ρ*^*c*^(*t*) and *ω*^*c*^ are the probabilities of being not sampled or not dying respectively, and *g*(*u*) is a stochastic function that maps from real-value *u* to integer *g*(*u*); it takes value ⌊*u*⌋ with probability *u* mod 1 and value ⌈*u*⌉ with probability 1 − (u mod 1). In plain English, the model tracks the random fate of each newly infected person as they move from the exposed to infected state with respect to eventually being observed as a case and/or a death in data. At each time step of length Δ*t*, the change in state space is given by,

$$\begin{array}{cc} C_{0}\left(t+\Delta t\right) & =C_{0}\left(t\right)+X_{3}+X_{4}-F_{t},\\ Y_{1}\left(t+\Delta t\right) & =Y_{1}\left(t\right)+F_{t},\\ D_{0}\left(t+\Delta t\right) & =D_{0}\left(t\right)+X_{2}+X_{4}-H_{1t},\\ D_{1}\left(t+\Delta t\right) & =D_{1}\left(t\right)+H_{1t}-H_{2t},\\ D_{2}\left(t+\Delta t\right) & =D_{2}\left(t\right)+H_{2t}-H_{3t},\\ D_{3}\left(t+\Delta t\right) & =D_{3}\left(t\right)+H_{3t}-H_{4t},\\ Y_{2}\left(t+\Delta t\right) & =Y_{2}\left(t\right)+H_{4t}, \end{array}$$

where *F*_*t*_|*C*_0_ is a random variate from $\text{Bin}(C_{0},1-\text{exp}\left(-\lambda_{Y_{1}}\Delta t\right))$, and *H*_*it*_ is a random variate from $\text{Bin}(D_{i-1},1-\text{exp}(-\frac{4}{21}\Delta t))$. *X*_*i*_ indicates the *i*^*th*^ element of Multinomial random variate defined above. The rate λ_*Y*1_ determines the rate at which persons who will be counted are counted (i.e. lower values of λ_*Y*1_ mean a longer delay in cases showing up in the data). The values of *Y*_1:2_ are set to zero at the beginning of every day such that they accumulate the simulated number of cases and deaths that occur each day. Both the transmission rate and detection probability are allowed to vary with time in the following way:

$$\begin{array}{cc} \chi (t) & =\{\begin{array}{cc} \chi_{0} & \text{if}\;t\leq t_{\chi }^{\left(1\right)},\\ \chi_{0}+\frac{\chi_{f}-\chi_{0}}{t_{\chi }^{\left(2\right)}-t_{\chi }^{\left(1\right)}}(t_{\chi }^{\left(1\right)}-t) & \text{if}\;t_{\chi }^{\left(1\right)}<t\leq t_{\chi }^{\left(2\right)},\\ \chi_{f} & \text{if}\;t>t_{\chi }^{\left(2\right)}, \end{array}\\ \rho (t) & =\{\begin{array}{cc} \rho_{0} & \text{if}\;t\leq t_{\rho },\\ \rho_{0}+\frac{\rho_{f}-\rho_{0}}{\left(t_{f}-20\right)-t_{\rho }}\left(t_{\rho }-t\right) & \text{if}\;t_{\rho }<t\leq \left(t_{f}-20\right),\\ \rho_{f} & \text{if}\;t>(t_{f}-20), \end{array} \end{array}$$

where *t*_*f*_ is the final time in the given dataset. In plain English, the transmission rate is constant up to some time, $t_{\chi }^{\left(1\right)}$, where it linearly increases or decreases to the value *χ*_*f*_ by time $t_{\chi }^{\left(2\right)}$. The model is constrained such that $t_{\chi }^{\left(2\right)}<t_{f}-20$, that is, the transmission rate must be constant for at least 20 days before the end of the data collection period. This constraint is in place to avoid overfitting the final transmission rate. The detection rate likewise has a linear increase or decrease beginning at time *t*_*ρ*_; however, the increase or decrease continues to a fixed point that is 20 days before the final datum. Variation in the detection rate (i.e. the probability that an infected case will be counted in a fixed interval) over the course of the epidemic can strongly bias estimates of the population growth rate (derivation for the exponential growth case in S1 File); not allowing the detection probability to change over time could lead to discordance in the case count and death count time series.

### Parameter estimation

We assume that the data are Negative Binomial-distributed, conditional on the simulated number of cases and deaths that occur in a given time interval, i.e. the number of cases in the *i*^*th*^ observation period has density NegBin(*Y*_1_, *ϵ*_1_) and the number of deaths in the *i*^*th*^ observation period has density NegBin(*Y*_2_, *ϵ*_2_). The Negative Binomial is parameterized such that the first argument is the expectation and the second is an inverse overdispersion parameter that controls the variance of the data about the expectation; as *ϵ*_*i*_ becomes large, the data model approaches a Poisson with parameter *Y*_*i*_. Both *ϵ*_1_ and *ϵ*_2_ were estimated from the data.

Parameter estimation had two distinct steps: model selection and computation of confidence intervals. In the model selection phase, the model was fit to the data using an iterated particle-filtering method, implemented in the pomp R library [8]. To optimize the likelihood of the data, we used 1500 particles in 125 iterations for each country. To determine whether or not 1500 particles was sufficient to minimize the variance in the estimated the mean likelihood at a given parameter value we ran 20 independent particles filters on a single data set and found that the average deviation from the mean was less than 0.1 log units suggesting that we would be able to detect differences in the log likelihood greater than 0.1 log units. The reported likelihoods were measured using 4500 particles at the optimized Maximum Likelihood Estimates (MLEs).

For all fits, the initial state at time zero is computed by assuming there were *I*(0) infected persons, 21 days before the first reported death (by definition time one). The number of initial number of susceptibilities was assumed to be the predicted 2020 population size of the given country [9]. The model is then simulated forward for 21 days, assuming exponential growth with transmission rate *χ*_0_, which is taken at the initial state of the model at time zero. Parameters have constraints *I*(0) ∈ [1, 1000], *ρ*_0_ ∈ [0, 1], *ρ*_*f*_ ∈ [0, 1], $\lambda_{Y1}^{-1}\in \left[5,21\right]$ *t*_*χ*_ and *t*_*ρ*_ ∈ [0, *t*_*f*_ − 10]; all other parameters are constrained to be positive.

Because the data were highly variable in the complexity of the patterns they showed, we considered three nested models of increasing complexity for each country. The first model (model 1) assumed simple exponential growth with a constant sampling probability, i.e. *ρ*_0_ = *ρ*_*f*_ and $t_{\chi }^{\left(1\right)}=t_{\chi }^{\left(1\right)}=0$. This amounts to a period of exponential growth with transmission rate *χ*_0_ in the pre-data period and then a constant transmission rate *χ*_*f*_ over the observation period. The second model (model 2) allowed the transmission rate to vary but kept the detection probability constant, i.e. *ρ*_0_ = *ρ*_*f*_. The third model (model 3) allowed both the transmission rate and detection rate to vary, i.e. all parameters estimated. We determined the best model for each country by a sequence of likelihood ratio tests first comparing model 1 and model 2 and then model 2 to model 3.

Because we are using an optimization method, we do not have access to samples from the likelihood surface directly. Therefore, to obtain estimates of the parametric uncertainly in the final transmission rate, *χ*_*f*_, we computed 99% confidence intervals using the profile likelihood method. For each country computed the profile likelihood by optimizing the model along a fixed grid with points every 0.1 units centered on the MLE of *χ*_*f*_ value from the previous fit. Points were added to the grid until the measured likelihood on either side of the MLE was greater than 9 log units lower than the measured maximum likelihood. We then fit a local polynomial regression (loess in R) to those points and found the predicted maximum likelihood parameter value [10] and the 99% CI by locating the points on either side of the MLE that were 3.84 log units below the maximum likelihood.

### Fixed parameter values

We fixed several parameter values based on published work and our scientific judgement. The probability that a case would eventually die, *ω* = 1%, is based on estimates of the case fatality ratio for both asymptomatic and symptomatic patients (95% CI 0.5-4%) [11]. The latency rate, $\lambda_{EI}=\frac{1}{3}$, was initial set based on our general sense of what was consistent with the pre-print literature available at the time, however, that value is has proven to be consistent with later reports [12]. The recovery rate, $\lambda_{IR}=\frac{1}{10}$, was similarly set to be consistent with the available literature when the model was being developed. Likewise, longitudinal studies have shown that our assumption of an average infectious period of 10 days is reasonably consistent with the clinical data [13, 14]. Although the clinical data paints a picture of the natural history of infection that is far more complex than our model can capture, the formulation of our model is consistent with the available data.

### Outcomes

Our primary outcome of interest is the growth rate, *r*, at the end of observation period, which is derived from the transmission rate estimated from the data. Using the equation in [15] we can express the growth rate in terms of the model parameters

$$\begin{array}{c} r=\frac{-\left(\lambda_{EI}+\lambda_{IR}\right)((\lambda_{EI}-\lambda_{IR}{{)}^{2}+4\lambda_{EI}\chi \text{Pr}\left(S\right))}^{\frac{1}{2}}}{2}. \end{array}$$

We found that for most countries, the fraction of the population that was still susceptible at the end of the observation period was greater than 95%, therefore we omitted the term $\text{Pr}\left(S\right)=\frac{S}{N}$ from the plots and tables to facilitate comparisons of changes in transmission and growth rates between countries. From the same paper we also have the equation

$$\begin{array}{c} R_{0}=\frac{\left(r+\lambda_{EI}\right)\left(r+\lambda_{IR}\right)}{\lambda_{EI}\lambda_{IR}} \end{array}$$

showing that *R*_0_ = 1 when *r* = 0.

We also compare predicted deaths due to COVID-19 in each country through July 5th 2020 to the average number of deaths in a period of the same length (Fig 3). Predicted deaths were computed by simulating the number of daily deaths from the first observation though July 5th 2020 for each country and taking the mean value. Confidence intervals were computed as the relevant quantiles of the sum over 1000 simulations. Pre-COVID-19 deaths were based on all-cause death data were downloaded from the WHO mortality database (https://www.who.int/healthinfo/mortality_data/en/) for each country for which it was available. Using data from the most recent year, we computed the death rate and multiplied the death rate by the length of the interval from the first observation to July 5th 2020 for each country.

## Results

**Fig 1:**
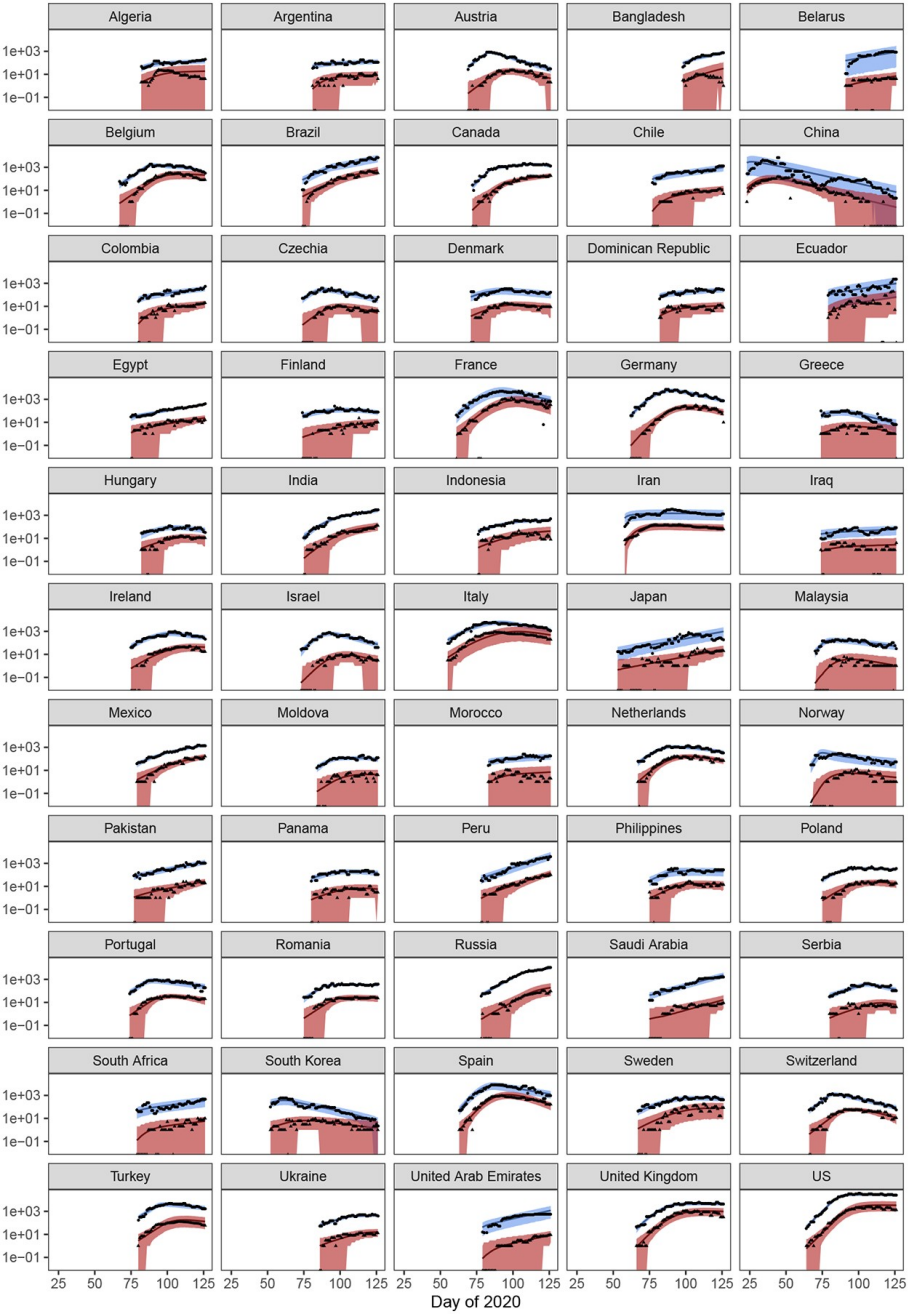
Model fits. Smoothed data points are shown in black and the expectations from the model are shown as solid lines. Model results for cases are shown in blue colors and deaths are shown in red. The envelope shows the 95% CIs of the distribution of the data conditional on the maximum likelihood model fit for the best model for each country. The x-axis is measured in sequential days of the year 2020.

We fit our model to data collected from 55 countries. Model fits are shown in Fig 1, and parameter values are given in Table 1. The model can capture the data well, with a few exceptions. The model was not able to find a robust fit to the data from Bangladesh; in general, the upside down ‘V’ shape to the deaths could not be captured well in such a short time series. Algeria had a similarly odd pattern in the deaths time series that the model could not capture in detail, although the overall trend in deaths was recovered. In previous versions of this paper, the model had a hard time fitting data from both Italy and Spain. However, given the longer time series and modifications to the model form, we now find that both Italy and Spain are well-captured by the model.

Overall, the model slightly overestimates the number of deaths around the time where deaths begin to decrease. However, this was generally corrected if the time series was long enough. Including temporal heterogeneity in the time from infection to detection of a COVID-19 death would likely correct this; however, it is not clear that this is advisable, as death counts are likely under-reported.

All countries except Japan and Saudi Arabia were found to have lower transmission rates on May 6, 2020 than at the beginning of the observation period, suggesting a global decline in the transmission of COVID-19 though May 6, 2020. However, the initial transmission rate should be interpreted cautiously, as we allowed a wide range of infected persons to exist 21 days before the first observation. That is, the initial transmission rate parameter is rather a convolution of the unknown number of infected persons and initial growth rate consistent with the data.

**Fig 2:**
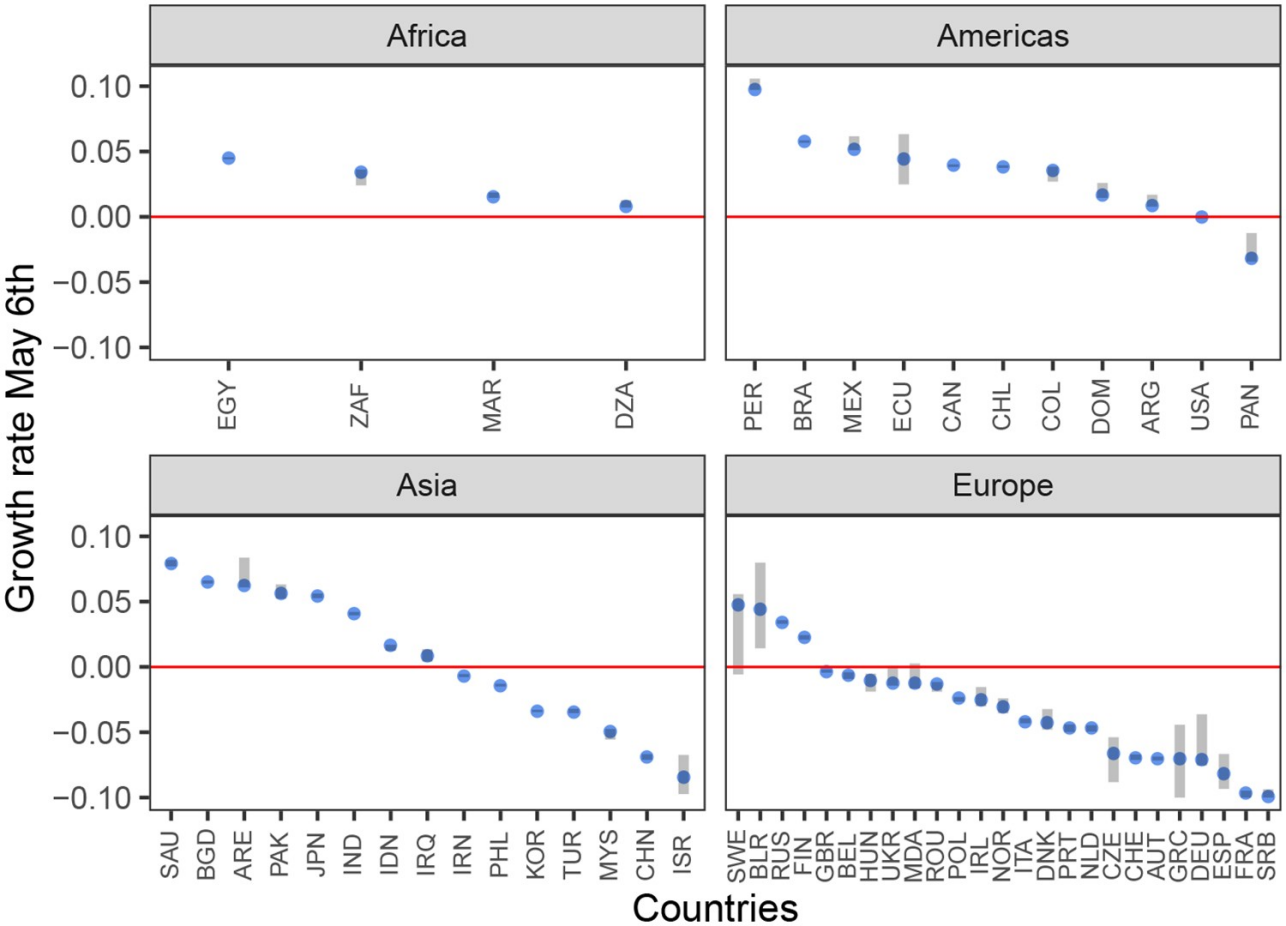
Values of the final exponential growth rates as of May 6 2020. Blue dots indicate point estimates and grey bars indicate the 99% confidence intervals. The red line delineates exponential growth from exponential decay. Countries below this line have a shrinking epidemic. Country names are indicated by ISO Alpha-3 codes.

We found significant evidence for subcritical dynamics in 27 countries (3 countries had subcritical point estimates but their CIs contained the epidemic threshold). Fig 2 shows the point estimates and CIs of the final transmission rate on May 6, 2020 for all countries, stratified by continent [16]. European countries had the highest probability of being subcritical (21 of 25) with Asia (7 of 15) and the Americas (2 of 11) having fewer subcritical countries. None of the countries in Africa that met the inclusion criteria had subcritical dynamics.

Generally, countries that were found to have both variable transmission rates and variable detection probabilities (model 3 in Table 1) show a pattern of level or increasing deaths coupled with a level or slightly declining incidence in number of reported cases. This pattern illustrates how viewing the case count data alone can be potentially misleading as declines in reported cases can be confounded by variation in the probability of detection (e.g. comparing Canada to Denmark). Some countries were found to have detection probabilities lower than 1% on May 6 2020; however, these values should not be over-interpreted as the simple linear model for changing detection probabilities imposes strong assumptions that are focused on capturing the general trend.

**Fig 3:**
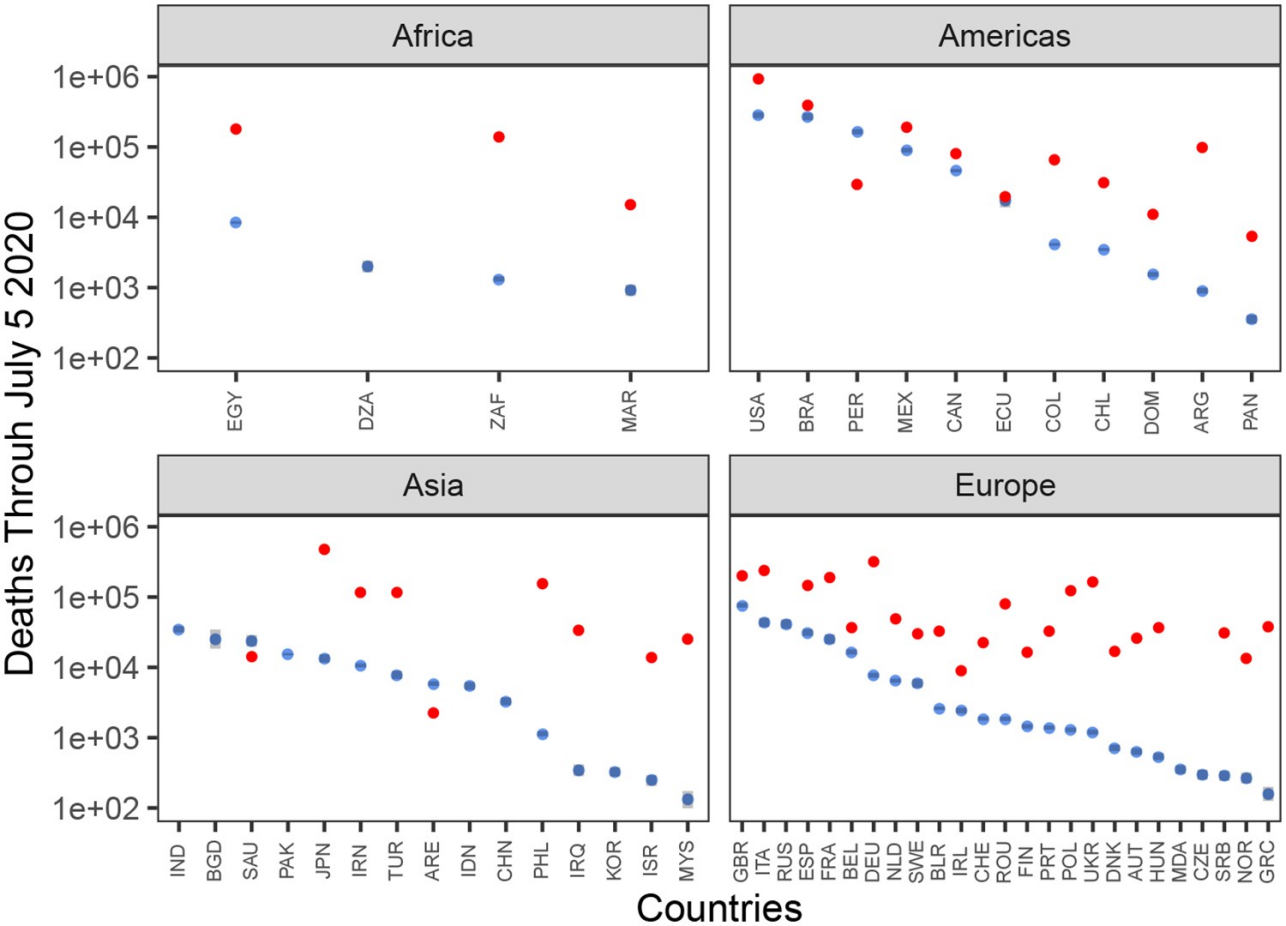
Predicted number of cumulative deaths through July 5 2020. The distribution of the total number of predicted deaths in the observation period plus 60 days assuming final model maximum likelihood values on May 6 2020 are constant though July 5 2020. Blue dots indicate median values and grey bars show the 95% confidence interval for the predicted number of deaths. Red dots show the average number of deaths in an average period of the same length in the World Health Organization death data from the most recent year available. The y-axis is on the log10 scale. Country names are indicated by ISO Alpha-3 codes.

Fig 3 shows the predicted number of deaths projected out to July 5, 2020 assuming that all parameter values are constant over the period May 6 through July 5 2020. The average duration of the country-level epidemic in European countries is longer than in Asia, leading to a higher level of death, despite Asian countries having on-average higher growth rates. However, in the Americas, the predicted deaths are higher with 8 of 11 countries having total predicted deaths greater than 0.1% of the total population by July 5, 2020. The model predicts 1,320,170 total COVID-19 deaths in all 55 countries on July 5, 2020; of those deaths, 21% are predicted to occur in the US. The deaths due to COVID-19 in Europe are lower than the average number of reported deaths in a period of the same length for all countries in the data set that also had all-cause death counts from previous years. However, in the Americas, the COVID-19 death counts are approaching the all-cause death levels in several countries, suggesting that COVID-19 deaths are approaching a doubling of all-cause deaths in those countries.

## Discussion

Our model found evidence for reductions in transmission rates of COVID-19 in 53 of 55 examined countries. Encouragingly, of those countries, we found statistical evidence that the size of the epidemic is decreasing in 27 countries, i.e. the effective reproductive number is less than 1, using data up to May 6, 2020. This suggests that, despite the highly heterogeneous populations represented by these countries, the growth of COVID-19 outbreak can be reverted. Although our model cannot attribute exact causes to the global decline in transmissions rates, most countries implemented sustained, population-level social distancing efforts over a period of weeks to months. These efforts are highly likely to play a major role in reducing the transmission of COVID-19 [5].

We estimated that in countries with decreasing transmission, the rate of decrease is in general less than 0.1/day (average -0.04/day). Based on data from 8 European countries, the US, and China, we previously estimated that in the absence of intervention efforts, the epidemic can grow at rates between 0.19-0.29/day [2]. This means that the outbreak can grow rapidly and quickly wipe gains made though public heath efforts made if social distancing measures are completely relaxed. For example, if the rate of decrease under strong public health interventions is 0.1/day and the growth rate in the absence of public heath interventions is 0.2/day, then, the number of cases averted in two weeks of intervention will be regained in only one week. Social distancing measures have their own social costs. Our results suggests alternative strategies to control the virus are needed in place when social distancing efforts are relaxed. Due to the uncertainties in the impact of each specific control measures, changes to policies should be made slowly because the signal of changing transmission can take weeks to fully propagate into current data streams as a result of the long lag between infection to case confirmation (as we estimated to be on average approximately 2 weeks).

Our goal in this paper was to develop a model that could be applied very broadly to multiple countries and we have made assumptions that facilitate that goal. However, our model makes key assumptions that should be considered when thinking about where these results fit into the vast collection of COVID-19 modeling papers. For example, we slightly privilege death counts over case counts in linking the population model to the data by assuming that the distribution of the time to death is known and that the probability of being a detected death is fixed over time. Likewise, we assume that at the country level, the change in transmission rates can be modeled by a simple linear function, which we believe is reasonable as interventions implemented at the local level are likely to lead to a smooth change when aggregated up to the population level. Our model produces reasonable fits to the global data, but out approach does not allow us to have unique models for each country that could almost certainly capture country-level trends with greater accuracy.

Our model also makes strong simplifying assumptions about the natural history of infection, about which we are continuously learning. For example, our model assumes that contagiousness is constant over the infection, which is possibly variable over time [12]. We also assume that diagnosis does not affect transmission from infected persons. However, given that we are inferring broad, population-average parameters and allowing those parameters to change over time to reflect broad changes in the transmission dynamics, we believe that our results are are reasonable portrayals of reality despite using a simple model of the natural history of infection.

Overall, our results suggest that COVID-19 is controllable in diverse settings using a full range of strong and comprehensive non-pharmaceutical measures, and that future deaths from the disease are avoidable.

## Supporting information

S1 File (PDF)
