# Supplementary material for: Change in global transmission rates of COVID-19 through May 6 2020
Source: PLoS One. 2020 Aug 6;15(8):e0236776. doi: 10.1371/journal.pone.0236776 (PMC7410207; doi:10.1371/journal.pone.0236776)
Supplement: S1 File — (PDF) [file pone.0236776.s001.pdf]

Supplemental

Estimating the infection and counting rates with exponential growth

Consider an exponential growth model early in the outbreak where infected (I) persons transmit at rate β, are counted (C) at rate $\alpha(t)$, recover (R) at rate γ_1, and die (D) at rate γ_2. When individuals are counted, they are not removed from I and transmit at the same rate as uncounted persons. The differential equations for I and C cases in time t, where (I, C, R, D) are dimensionless numbers, but $(\alpha, \beta, \gamma_1, \gamma_2)$ have units of $(\text{time})^{-1}$, state,

$$\frac{dI(t)}{dt} = \beta I(t) - \gamma_1 I(t) - \gamma_2 I(t), \quad (1)$$

$$\frac{dC(t)}{dt} = \alpha(t) I(t), \quad (2)$$

$$\frac{dR(t)}{dt} = \gamma_1 I(t), \quad (3)$$

$$\frac{dD(t)}{dt} = \gamma_2 I(t). \quad (4)$$

Let us also tie C to R. We have that,

$$\frac{C(t)}{dt} = \alpha(t) I(t), \quad (5)$$

$$= \frac{\alpha(t)}{\gamma_2} \frac{dD(t)}{dt}. \quad (6)$$

The problem is that we only observe $dC(t)/dt$ and $dD(t)/dt$. So algebraically,

$$\alpha(t) = \gamma_2 \frac{dC(t)/dt}{dD(t)/dt}. \quad (7)$$

Given the model specified, it is true that $\alpha = \gamma_2 (dC/dt)/(dD/dt)$. Since they are total derivatives, it is also true that $\alpha = \gamma_2 dC/dD$.

Constant counting rate

It is a first-order problem to show that,

$$I = I_0 \exp\left[(\beta - \gamma_1 - \gamma_2)t\right], \quad (8)$$

$$S = -\beta(\beta - \gamma_1 - \gamma_2)^{-1} I, \quad (9)$$

$$R = \gamma_1(\beta - \gamma_1 - \gamma_2)^{-1} I, \quad (10)$$

$$D = \gamma_2(\beta - \gamma_1 - \gamma_2)^{-1} I, \quad (11)$$

as well as, when $d\alpha/dt = 0$,

$$C = \alpha(\beta - \gamma_1 - \gamma_2)^{-1} I. \quad (12)$$

An interpretation is that $I(t) = C(t + \tau)$, with time lag of τ applied to t, where,

$$\tau = \frac{1}{\beta - \gamma_1 - \gamma_2} \log\left(\frac{\alpha}{\beta - \gamma_1 - \gamma_2}\right). \tag{13}$$

This simply shows that if the counting rate is constant, then the value of C is simply equal to I shifted in time by τ.

Time-varying counting rate

For time-varying α, at time t with initial count C_0 at time t_0, in terms of (the only quantities observable) C & D,

$$\begin{aligned} C &= C_0 + (\gamma_2)^{-1} \int_{t_0}^{t} \alpha dD, & (14) \\ &= C_0 + (\gamma_2)^{-1} \left[\alpha(t)D(t) - \alpha(t_0)D(t_0) - \int_{t_0}^{t} D(t')(d\alpha/dt')dt'\right]. & (15) \end{aligned}$$

Stipulating that $C_0 = (\gamma_2)\alpha(t_0)D(t_0)$, up to an additive constant,

$$C = (\gamma_2)^{-1} \left[\alpha(t)D(t) - \int_{t_0}^{t} D(t')(d\alpha/dt')dt'\right], \tag{16}$$

which reduces back, if $d\alpha/dt = 0$, to the time-integral of Equation 7:

$$C = (\gamma_2)^{-1}\alpha D(t). \tag{17}$$

Exponential-growth difference between counts and infections

In the case where $d\alpha/dt > 0$, we find the difference $(d \log C)/dt - (d \log I)/dt$. It is generally true that,

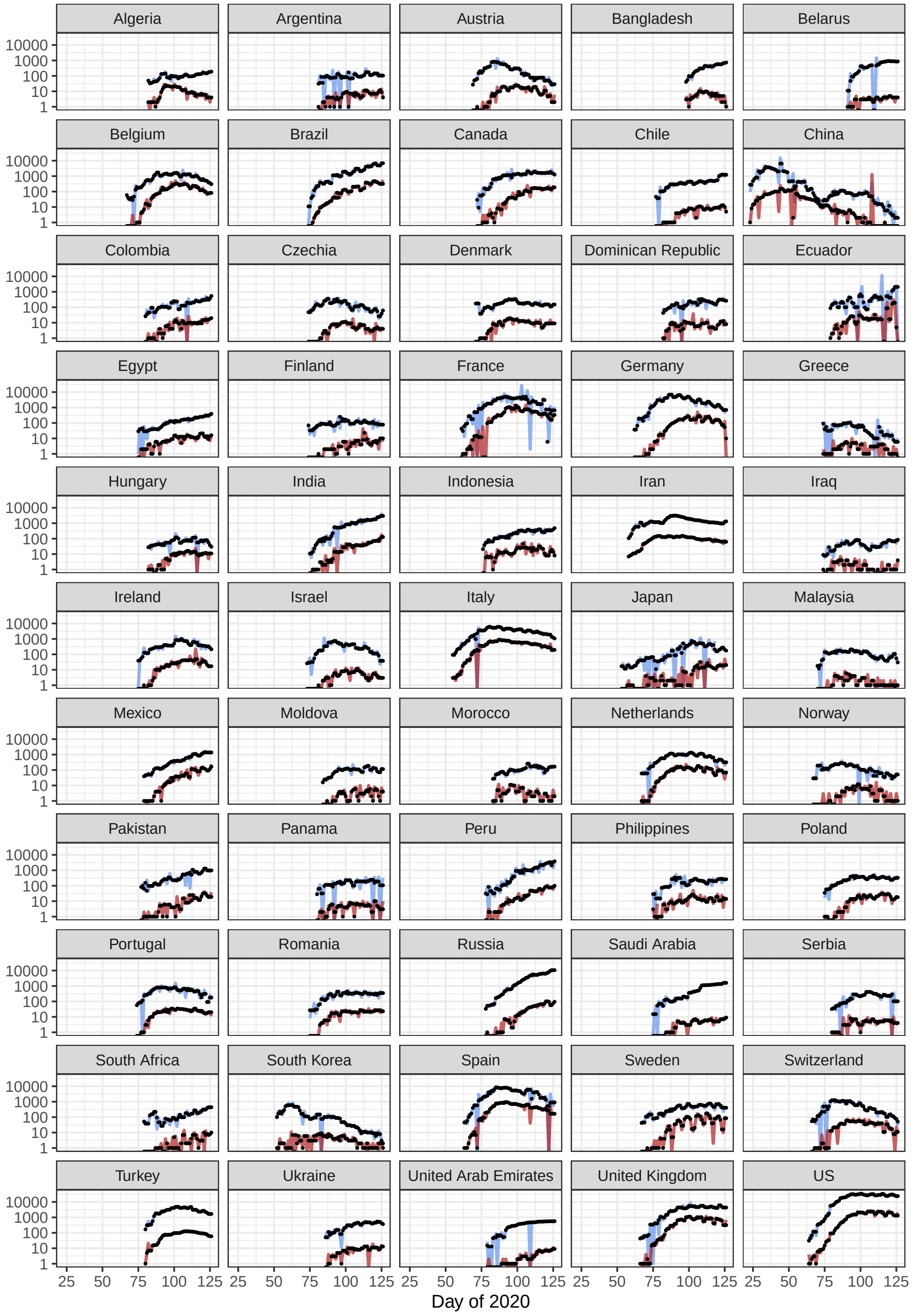

S1 Fig. Raw and smoothed data. Raw data for cases (blue lines) and deaths (red lines) are shown with the smoothed time series (black) used for model fitting. The x-axis is measured in sequential days of 2020.

$$\frac{dI}{dt} = \alpha^{-1}(\beta - \gamma_1 - \gamma_2)\frac{dC}{dt}. \tag{18}$$

Using $dx = xd\log x$ to transform C, I,

$$\frac{d\log I}{dt} = \frac{C}{I}\alpha^{-1}(\beta - \gamma_1 - \gamma_2)\frac{d\log C}{dt}. \tag{19}$$

Yet integration by parts shows that, for general $d\alpha/dt$,

$$C = (\beta - \gamma_1 - \gamma_2)^{-1}\left[\alpha(t)I(t) - \int_{t_0}^{t} I(t')(d\alpha/dt')dt'\right], \tag{20}$$

substitution of which yields,

$$\frac{d\log I}{dt} = \left(1 - \frac{1}{\alpha(t)I(t)}\int_{t_0}^{t} I(t')(d\alpha/dt')dt\right)\frac{d\log C}{dt}, \tag{21}$$

Whenever $d\alpha/dt$ is positive and, as almost always the case, $\alpha, I, dI/dt > 0$, then Equation 21 implies that $\log C$ grows faster than $\log I$. In the approximation that α changes slowly,

$$\int I(d\alpha/dt')dt' \ll \alpha(t)I(t),$$

then Taylor expanding and rearranging terms yields a simpler expression,

$$\frac{d\log C}{dt} - \frac{d\log I}{dt} \approx \left(\frac{1}{\alpha(t)I(t)}\int_{t_0}^{t} I(t')\frac{d\alpha(t')}{dt'}dt'\right)\frac{d\log I}{dt}, \tag{22}$$

$$= \frac{1}{\alpha(t)e^{(\beta-\gamma_1-\gamma_2)t}}\int_{t_0}^{t} e^{(\beta-\gamma_1-\gamma_2)t'}\frac{d\alpha(t')}{dt'}dt'. \tag{23}$$

For all positive $\alpha, d\alpha/dt$, the right-hand side is positive. Evidently, the growth rate in logarithmic C exceeds that in logarithmic I. The growth rate of logarithmic C can be readily inferred from a log-linear plot, but I is generally unknown. When the testing rate increases during an epidemic's exponential growth phase, the number of counts C increases faster than the number of infections I.
